# Remote Tracking Gas Molecular via the Standalone-Like Nanosensor-Based Tele-Monitoring System

**DOI:** 10.1007/s40820-020-00551-w

**Published:** 2021-01-04

**Authors:** Han Jin, Junkan Yu, Daxiang Cui, Shan Gao, Hao Yang, Xiaowei Zhang, Changzhou Hua, Shengsheng Cui, Cuili Xue, Yuna Zhang, Yuan Zhou, Bin Liu, Wenfeng Shen, Shengwei Deng, Wanlung Kam, Waifung Cheung

**Affiliations:** 1 Institute of Micro-Nano Science and Technology, School of Electronic Information and Electrical Engineering, Shanghai Jiao Tong University, Shanghai, 200240 People’s Republic of China; 2 National Engineering Research Center for Nanotechnology, Shanghai, 200240 People’s Republic of China; 3 School of Electrical Engineering and Computer Science, Ningbo University, Ningbo, 315211 People’s Republic of China; 4 State Key Laboratory of Pathogen and Biosecurity, Institute of Microbiology and Epidemiology, Academy of Military Medical Sciences, Beijing, 100071 People’s Republic of China; 5 Ningbo Materials Science and Technology Institute, Chinese Academy of Sciences, Ningbo, 315201 People’s Republic of China; 6 College of Chemical Engineering, Zhejiang University of Technology, Hangzhou, 310014 People’s Republic of China; 7 Qi Diagnostics Ltd, Hongkong, People’s Republic of China

**Keywords:** Metal–organic framework-derived polyhedral ZnO, Perovskite quantum dots, Nanosensor, NO_2_, Tele-monitoring system

## Abstract

**Highlights:**

- A standalone-like smart device that can remotely track the variation of air pollutants in a power-saving way is created;
- Metal–organic framework-derived hollow polyhedral ZnO was successfully synthesized, allowing the created smart device to be highly selective and to sensitively track the variation of NO_2_ concentration;
- A novel photoluminescence-enhanced Li-Fi telecommunication technique is proposed, offering the created smart device with the capability of long distance wireless communication.

**Abstract:**

Remote tracking the variation of air quality in an effective way will be highly helpful to decrease the health risk of human short- and long-term exposures to air pollution. However, high power consumption and poor sensing performance remain the concerned issues, thereby limiting the scale-up in deploying air quality tracking networks. Herein, we report a standalone-like smart device that can remotely track the variation of air pollutants in a power-saving way. Brevity, the created smart device demonstrated satisfactory selectivity (against six kinds of representative exhaust gases or air pollutants), desirable response magnitude (164–100 ppm), and acceptable response/recovery rate (52.0/50.5 s), as well as linear response relationship to NO_2_. After aging for 2 weeks, the created device exhibited relatively stable sensing performance more than 3 months. Moreover, a photoluminescence-enhanced light fidelity (Li-Fi) telecommunication technique is proposed and the Li-Fi communication distance is significantly extended. Conclusively, our reported standalone-like smart device would sever as a powerful sensing platform to construct high-performance and low-power consumption air quality wireless sensor networks and to prevent air pollutant-induced diseases via a more effective and low-cost approach.
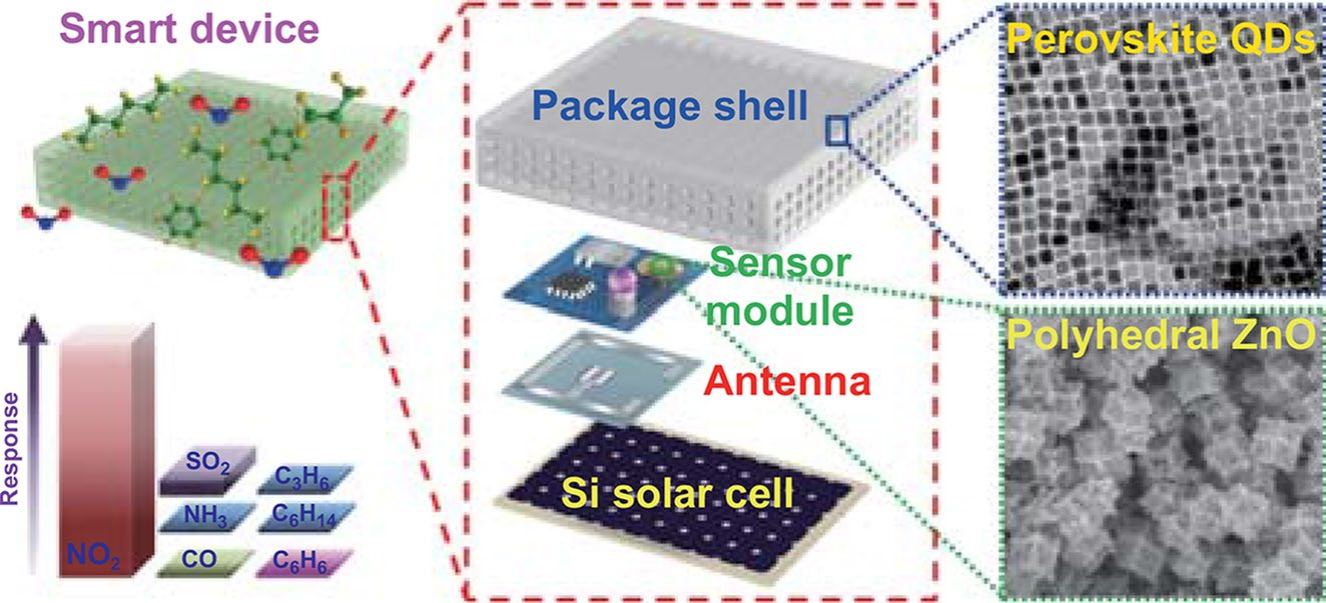

**Electronic supplementary material:**

The online version of this article (10.1007/s40820-020-00551-w) contains supplementary material, which is available to authorized users.

## Introduction

Excessive use of fossil fuels leads to a dramatic increase in the emission of air pollutants (e.g., NO_2_, SO_2_, and CO) [1]. These air pollutants result in both acute and chronic effects on human health, including but not limited to chronic respiratory, heart disease, lung cancer, and asthmatic attacks in both children and adults [2, 3]. For instance, it is widely agreed that air pollutants such as NO_2_ and SO_2_ can affect human airway epithelial cells through inducing a greater response in genes associated with oxidative stress [4, 5]. While, high level of NO_2_ may increase the risk for developing acute exacerbation of chronic obstructive pulmonary disease [6]. In order to effectively evaluate the air quality, air pollution index (API) or the air quality index (AQI) is recently set to indicate how serious the air pollution is [7]. Citizens can easily access the value of API/AOI via internet, and sensitive populations (*e.g.,* children, the elderly and patients with heart/respiratory illness) could possibly avoid these adverse effects derived air pollutants through reasonably planning their daily outdoor activities [8]. Herein, tracking the variation of air quality in an effective way will be highly helpful to decrease the health risk of short- and long-term exposures to air pollution.

In the past couple of decades, stationary monitoring stations were firstly employed to collect the data of API/AQI [9]. These stations were typically featured with highly accurate data, but measurement equipment that employed in these stations was also typically very expensive, thereby limiting their scale-up in deploying API/AQI tracking networks [10]. In comparison with those expensive stationary monitoring stations, wireless sensor node demonstrates the advantage of cost-effective and low-energy consumption as well as simple configuration [10, 11]. Furthermore, wireless sensor networks (WSNs) that consist of a number of air quality sensor nodes hold the potential to increase the achievable spatial density of measurements [11, 12]. In light of these merits, there has been a growing interest in the development and deployment of WSNs that employ smart air quality sensors. A typical example is that China has deployed air quality WSNs in major cities (e.g., Shanghai and Beijing) [2, 3]. With the help of wireless communicating technology, such as ZigBee, Wibree, and Sigfox, air quality in the WSNs deployed cities can be remotely tracked [1, 12–14]. Generally, high-performance tracking the level of air pollutants and operating at power-saving mode are two basic criteria for designing the WSNs so that each sensor node is able to effectively monitor the variation of air pollutants and to keep working for a long period without charging or replacing the battery [14]. A promising way to decrease the power consumption is to adopt an energy harvesting strategy (e.g., nanogenerator or solar cell) to the monitoring system [15–20]. In addition, since the sensing materials and the wireless telecommunicating technology directly determine the sensing characteristics and power consumption, the development of high-performance sensor and low-power telecommunicating technology is highly desirable.

Over the past period, nanotechnology has been adopted to promote the sensing characteristics of the air quality sensors [21–24]. Particularly, the use of various nanomaterials essentially enhanced the selectivity and sensitivity [21, 23]. While, emerging of light fidelity (Li-Fi)-based telecommunicating technology allows the data to be delivered in a more power-saving way when compared with the frequently reported ZigBee or WiFi [25]. Based on this description, it is envisioned that the combination of nanotechnology and Li-Fi telecommunicating technique would provide an efficient approach to design air quality-oriented smart WSNs. Herein, we reported a standalone-like smart device to serve as the air quality sensor node in which a MEMS (microelectro mechanical systems) nanosensor is designed to selectively and sensitively detect air pollutants (e.g., NO_2_). A Si solar cell that is contained in the reported device allows the energy to be harvested from the sunlight. Moreover, photoluminescence-enhanced Li-Fi telecommunicating technology is proposed and adopted to deliver informative data. With the assistance of a high-resolution camera, Li-Fi signal can be remotely captured and uploaded to the server for further evaluating the air quality. In sum, a demo of promoting nanotechnology and cutting-edge telecommunicating technology in designing future smart device for specific environmental application is demonstrated in this article.

## Experimental Section

Materials synthesis details can be found in the flow chart shown in Fig. S1.

### Synthesis of Perovskite Quantum Dots and Materials Characterization

All-inorganic CsPbX_3_ (*X* = Cl, Br, I) perovskite quantum dots (QDs) were synthesized via a conventional hot-injection approach. Firstly, Cs_2_CO_3_ (1.628 g) was loaded into a 200-mL flask which contains a mixture of octadecene (80 mL, ODE) and oleic acid (5 mL, OA), stirred and heated under vacuum to 200 ºC until all Cs_2_CO_3_ reacted with OA. ODE (10 mL), OA (1 mL), oleylamine (1 mL, OLA), and PbX_2_ (0.376 mmol, *X* = Cl, Br and I) were mixed in a 50-mL flask and dried under vacuum at 120 °C for 1 h. OLA and OA were injected at 120 °C under N_2_ atmosphere. Then, the temperature was raised to 160 °C and 0.4 mL Cs-oleate solution was rapidly injected. After 5 s, the solution was cooled by the water bath. All chemical products were used as received without further purification. Stead-state photoluminescence (PL) emission spectra are obtained by using the Horiba Jobin–Yvon Fluorolog-3 system equipped with a 30-mW He-Cd laser as the excitation source. The Hamamatsu R928 photomultiplier tube (PMT) is used for luminescent detection. Absorption spectra are measured by using the Shimadzu UV3600 spectrometer. All the spectra are corrected for the system response. Microstructure of all-inorganic CsPbBr_3_ QDs is confirmed by using the FEI TECHNAI-F20 field emission transmission electron microscope (TEM) operated at 200 kV.

### Synthesis of Metal–Organic Framework-Derived Porous ZnO and Materials Characterization

2.20 g Zn(NO_3_)_2_·6H_2_O and 4.87 g C_4_H_6_N_2_ were dissolved into 25 mL methanol and stirred at room temperature for 5 h. Then, the product was collected by centrifugation and washed with ethanol for several times; Metal–organic framework (MOF) precursor was recovered by drying the powder at 50 °C overnight. Finally, the porous ZnO was obtained by calcining the MOF precursor at 450 °C for 4 h. The crystal phase, microstructure, and surface area, as well as the pore diameter, are characterized by means of X-ray diffraction analysis (Rigaku Ultima IV, Japan; Cu Ka radiation, *λ* = 1.5418 Å), FESEM (Hitachi SU5000, Hitachi Corp., Japan), TEM (FEI Tecnai G2 f20 s-twin, 200 kV), BET, and pore diameter (ASAP 2020, Micromeritics, USA).

### Sensor Fabrication and Sensing Performance Evaluation

MOF-derived ZnO was separately ground with several drops of ethanol to form a dilute paste, which was dropped onto a photolithography made sensor chip with a pair of interdigital electrodes and a pair of active heater (HHC1000, Hefei Nano-micro Sensing Tech. company, China). Note that, prior to coating the presented sensing material, the photolithography-made sensor chip was pre-treated via oxygen plasma for several times to avoid the frequently meet coating fade off during testing. The temperature was controlled by tuning the heating voltage. Before the first measurement, the sensor was dried for one day at room temperature and then aged at 200 °C for 12 h. The response signal is defined as *S* = (*R*_sample gas_ − *R*_a_)/*R*_a_, where *R*_sample gas_ and *R*_a_ are the resistance of the sensor exposed to sample gas (100 ppm CO, NH_3_, NO_2_, SO_2_, C_6_H_6_, C_6_H_14_, C_3_H_6_, diluted with air) or air, respectively.

### Fabrication and Characterization of the Antenna

The antenna was fabricated by chemical vapor deposition (CVD). The radiation pattern of the antenna was measured by using a spherical multi-probe antenna near-field measurement system. The antenna is located in the center of the system on top of a foam column. The full sphere measurement is performed by electronically scanning the probe array in elevation and rotating the antenna in azimuth.

### Implementing the Li-Fi Communication

A commercialized high-resolution camera (OSG030-815UM, YVision, China) was used to collect the signal transmitted by the smart device. When response signal is generated, the generated decimal response signal was automatically converted to binary code. Binary codes were sent in the manner of Li-Fi. After the binary code is captured by the camera in the form of recorded optical images, images are further analyzed by the image recognition algorithm. Typically, two pixels in the recorded image, i.e., one from the smart device and one from the surrounding environment, are selected for comparison. If the absolute difference of the value for the selected two pixels is larger than a certain threshold, we regard it as “light on” (binary code = 1). Otherwise, it is defined as “light off” (binary code = 0). Note that the threshold of gray scale is set as 120 in this research [26]. In this case, environmental interference can be effectively eliminated. The camera records the image at the speed of 55 times per second and the initial binary code (at the beginning 45 s) sent by the nanosensor-based electronic is artificially set as 1. These 45 times consecutive 1 are regarded as identifying code. After capturing the initial identifying code, the received bits are identified as the effective signal. Finally, all the collected effective signal that appears in the form of binary code is converted to decimal number, so as to restore the original response signal.

## Results and Discussion

### Overall Strategy of Creating the Standalone-Like Smart Sensing Device

**Fig. 1:**
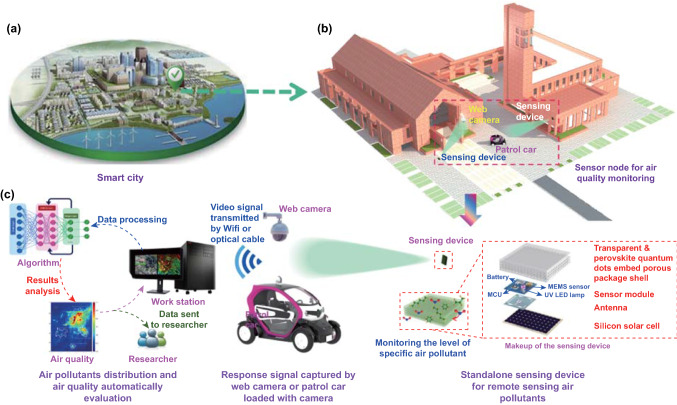
Illustration of the overall experimental strategy: **a, b** WSNs deployed city for remote tracking air quality. The standalone-like smart sensing device serves as the sensor node for real-time detecting the level of air pollutants; c. Smart sensing device with compact configuration in which all functional units were integrated in a 4 × 4 × 2 cm^3^ transparent box is designed in this research. With the assistance of photoluminescence-enhanced Li-Fi telecommunicating technology, informative signal can be remotely captured by web camera or the camera loaded patrol car. Followed by signal back end processing, the air quality in specific location of the city would be remotely assessed

To address the challenge of remote tracking the variation of air quality in a cost-effective and power-saving approach (Fig. 1a, b), a standalone-like smart device with the geometry of 4 × 4 × 2 cm^3^ (length × width × height) is created (Fig. 1c). Basically, the device contains the following functional units: (i) a transparent and perovskite QDs embed porous package shell is designed to enable the gas molecular of air pollutants reaching the surface of the sensor and to extend the Li-Fi telecommunicating distance; (ii) sensor module that comprised a MEMS nanosensor, LED lamp, mini battery, and the microcontroller unit (MCU, stm32F031F6P6, ST company, Italy) is fabricated to track the level of specific air pollutants and transmit signal in terms of Li-Fi; (iii) an internal antenna is designed to operate the sensing device at the intermittent/awake operation mode; (iv) Si solar cell for energy harvesting. Integrating with these abovementioned functional units, the smart device is expected to realize the vision of highly selective and sensitive sensing of the concerned air pollutants and transmitting the signal to headquarters with low-power consumption.

For the purpose of deploying WSNs that based on the created smart device in a more convenient way, the device is designed to harvest energy from sunlight in the daytime and storing the energy in the mini battery (120 mAh). Besides, with the intention of minimizing the power consumption, the smart device is designed to operate for 15 min per time, with the intervals of 2 h (hereinafter denoted as intermittent operation mode). In other words, the smart device stays at hibernate mode for most of the time. Since hibernate mode does not consume extra energy, it is speculated that the harvested energy during daytime could be enough to support the device discontinuously working for 180 min per day (15 min/time × 24 h/2 h). This would enable the smart device to be free of battery replacement during its whole service life, namely, the smart device can be operated under the standalone mode. Note that the whole device should be absolutely operated at low-power mode since limited energy (220 mA) would be supplied by the Si solar cell with the geometry of 4 × 4×0.2 cm^3^ (length × width × height). To realize the target, power-saved MEMS nanosensor that consists of the tailor-made nanomaterial is adopted to air pollutants tracking. Additionally, sensing performance of the MEMS nanosensor is photochemically activated to achieve high sensitivity through simply illuminating with a mini LED lamp.

Regarding data transmission, Li-Fi communicating technique is employed in the research. Brevity, the decimal response signal generated by the MEMS nanosensor will be quickly converted to binary code (0/1) and transmitted in the manner of Li-Fi. In this research, the mini LED lamp also serves as the signal transceiver to implement the Li-Fi-based data transmission. Basically, the LED lamp operated at the state of “light on” represents the binary code of 1 while “light off” represents the binary code of 0 and the binary code in the manner of optical image will be captured by a camera. Since the power consumption of the MEMS nanosensor and LED lamp is roughly estimated to be 30 and 680 mW, respectively, the total power consumption of the whole smart device is expected to be controlled within 850 mW which can be fully supported by the Si solar cell charged mini battery. To extend the communicating distance of the Li-Fi telecommunicating under sunshine, photoluminescence-enhanced Li-Fi telecommunicating technology is developed and the technique details will be elaborated in the following section.

### Deign and Creating the Smart Device to Achieve the Expected Electronic Function

**Fig. 2:**
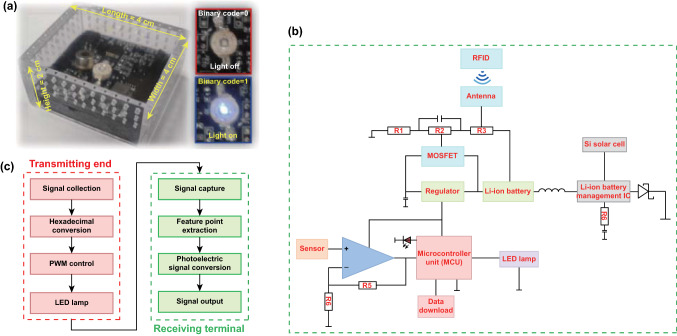
Device and driving circuit: **a** Photograph of the smart device. A LED lamp that integrated into the smart device is employed to implement the Li-Fi communication, when the LED light operated at light on is defined as binary code of “1,” while at light off is defined as binary code of “0”; **b** Schematic view of the circuit diagram to implement intermittent/awake operation mode and Li-Fi-based telecommunication. The intermittent/awake operation mode and Li-Fi-based telecommunication would minimize the power consumption of the created device; **c** Algorithm flow chart of the Li-Fi telecommunication

Photograph of the created standalone-like smart device and its Li-Fi communicating details can be found in Fig. 2a. Additionally, photographic images of all these functional units that integrated into the smart device are shown in Fig. S2. Figure 2b reveals the corresponding circuit diagram. Basically, the electrical deign of the circuit board shown in Fig. S2 involves the modules of RFID controller (triggering the battery switch through the near-field communication), power management (energy supply and battery life extends), signal amplification, microcontroller (signal gathering and converting), and direct current (DC)/alternative current (AC) boost converter (data transmission). The algorithm flow chart of the Li-Fi-based communication is shown in Fig. 2c and the detail of the program code can be found in Fig. S3 and Supplementary document of codes for Li-Fi telecommunication. The principle of the Li-Fi based communication can be briefly described as follows: response signal will be generated by the device after exposed to the ambient NO_2_ and automatically converted to binary code. Managed by the circuit board shown in Fig. 2b, binary codes would be transmitted through Li-Fi by quickly turn on/off the LED lamp (Fig. 2a). After the optical image-like binary code is captured by the camera, images will be restored to the original decimal response signal and further analyzed by data process terminal. Finally, the level of the air quality can be remotely assessed.

It should be particularly noted that beyond the intermittent operation mode, awake mode is additionally designed by integrated an antenna inside the device. When a patrol car loaded with a camera and a radio frequency identification (RFID) reader (Fig. S4a) reaches to the smart device within a certain distance (less than 7 m), the smart device that integrated with an antenna will be able to instantly awake from the hibernate state (Fig. S4b). In this case, the patrol car acts as the mobile web camera to capture signal. This would be useful to realize the data transmission if the smart device is placed at some locations without web camera. Technical parameter of the antenna is summarized in Fig. S5. In summary, a quasi-omnidirectional radiation pattern that would be similar to a conventional dipole antenna is observed which can be confirmed by the results of simulated reflection coefficient and 3D radiation pattern of the antenna (Fig. S4). The identical behavior for the simulated and measured results directly indicates the success of implementing the awake mode for the created smart device. Figure S4c, d gives the demonstration of the smart device operated at the awake mode. In conclusion, the designed circuit and implanted algorithm promote the original intention—power saving close to reality.

### Realizing the Photoluminescence-Enhanced Li-Fi Telecommunicating Technique

**Fig. 3:**
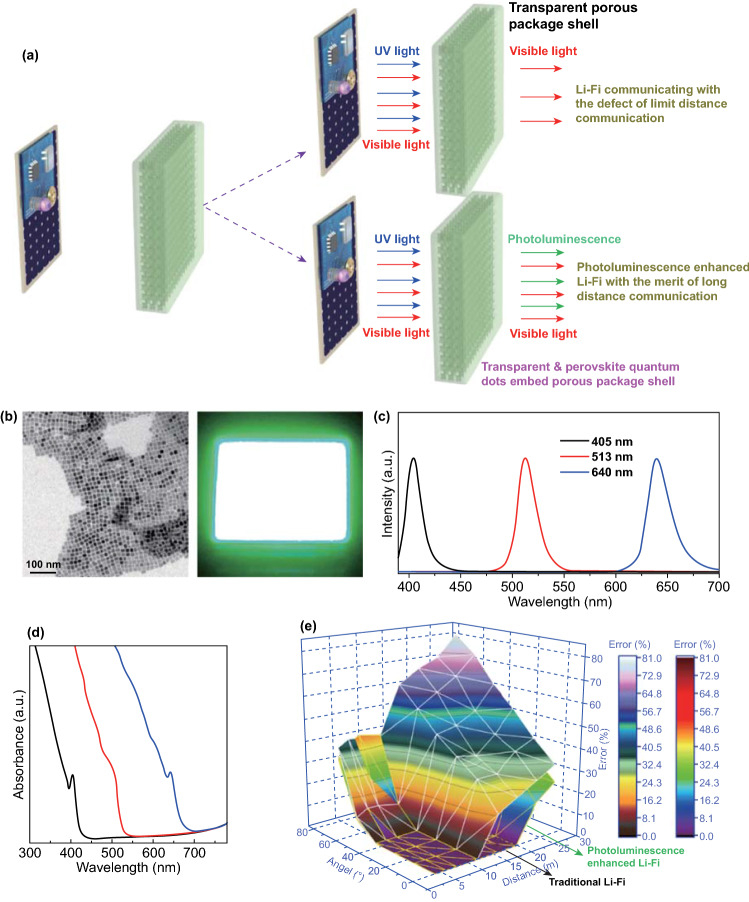
Characterization of the photoluminescence-enhanced Li-Fi telecommunication: **a** Illustration of the photoluminescence-enhanced Li-Fi telecommunicating technique; **b** TEM image of the CsPbCl3 QDs and its photoluminescence under the 365 nm illumination; **c** PL emission spectra of colloidal perovskite QDs; **d** absorption spectra of colloidal CsPbCl_3_, CsPbBr_3_, and CsPbI_3_ QDs; **e** Error analysis for the camera captured signal, at the viewing angle of 0-80° and the viewing distance of 0-30 m. The photoluminescence-enhanced Li-Fi essentially extended the communication distance and maintained the accuracy of the transmitted data within the viewing angle of 45° and viewing distance of 20 m

Li-Fi telecommunication is adopted to wirelessly transmit the data in this research; however, a key problem, namely the short communication distance, should be figured out prior to employ the technique for signal transmission. The communication distance of the Li-Fi is directly determined by light intensity of the min LED lamp and as shown in Fig. 3a that the acrylic made porous package shell would block most of the UV light (365 nm), resulting in limit communication distance. Although this issue can be addressed by using a LED lamp with higher intensity, it increases the power consumption and shortens the service life. An alternative strategy to overcome the difficulty without raising the power consumption is to develop a photoluminescence-enhanced Li-Fi telecommunicating technique (shown in Fig. 3a). The principle of the photoluminescence-enhanced Li-Fi telecommunicating technique is as follows: a CsPbX_3_-based perovskite QDs layer was pre-coated on the inter-surface of the porous package shell. When illuminated by the LED lamp, perovskite QDs layer absorbs the UV light and generates visible photoluminescence. Consequently, light intensity can be significantly increased, extending the Li-Fi communication distance. Herein, CsPbX_3_-based perovskite QDs is employed since their photoluminescence spectra cover the area of visible light. The morphology of the CsPbX_3_-based perovskite QDs is investigated by TEM. In consideration of similar morphology for the CsPbX_3_ (*X*: Cl, Br, I) QDs, CsPbCl_3_ is selected as representative and its TEM image is shown in Fig. 3b. The monodisperse CsPbCl_3_ QDs shown in Fig. 3b demonstrated a typical cubic/cuboidal shape. The thickness of the CsPbX_3_ on the substrate that is measured by spectroscopic ellipsometer (Semilab SE-2000) is around 173 nm. Besides, luminescent image with high intensity is observed for the CsPbCl_3_ QDs illuminated by 365 nm UV lamp which will be helpful to extend the Li-Fi communicating distance. Figure 3c shows the photoluminescence (PL) emission spectra of colloidal CsPbCl_3_, CsPbBr_3_, and CsPbI_3_ QDs. The PL intensity peaks are located at 405, 513, and 640 nm, corresponding to the CsPbCl_3_, CsPbBr_3_, and CsPbI_3_ QDs, respectively. All synthesized perovskite QDs demonstrate relatively high quantum yields (54.5% for CsPbCl_3_, 76.8% for CsPbBr_3_ and 71.2% for CsPbI_3_, respectively) and narrow PL full width at half maximum (FWHM, 17 nm for CsPbCl_3_, 22 nm for CsPbBr_3_, 26 nm for CsPbI_3_, respectively). Figure 3d presents the absorption spectra of colloidal CsPbCl_3_, CsPbBr_3_, and CsPbI_3_ QDs, which suggests an excellent ability to capture UV radiation. Nevertheless, CsPbBr_3_ and CsPbI_3_ QDs also demonstrate the capability of the capturing part of visible light which may decrease the total light intensity. In contrast, CsPbCl_3_ only demonstrated the property of absorbing UV light, and thereby, a transparent and CsPbCl_3_ QDs-embed porous package shell is fabricated to realize the photoluminescence-enhanced Li-Fi telecommunication. The lifetime of the CsPbCl_3_ QDs' induced photoluminescence is at the order of nano-second (Fig. S6), indicating that the UV light-triggered photoluminescence will simultaneously disappear when LED lamp turns off. This means high precision in signal transmission is predicted for the photoluminescence-enhanced Li-Fi telecommunication.

Comparison on the error analysis for the photoluminescence-enhanced Li-Fi telecommunication and the traditional Li-Fi telecommunication (using the package shell without coating perovskite QDs) is analyzed under the sunlight. The measurement is carried out at the angle of 0–80° and the distance of 0–30 m. Thanks to the photoluminescence-enhanced Li-Fi telecommunicating technique and the image recognition algorithm, the camera successfully captured the Li-Fi transmitted signal without any error at the viewing angle of 0–45° and the viewing distance of 0–20 m, even measured under sunlight (Fig. 3d). However, the maximum communicating distance for tradition Li-Fi is about 13 m, with the viewing angle within the range of 0–45°. Significant deviation is observed when the distance and angle exceed the threshold value regardless telecommunication technique used for signal transmission (Fig. S7). It should be particularly noted that high-resolution camera and binary code-based communication play important role in Li-Fi-based telecommunication because of their powerful capability in image recording which is useful for the following image recognition. Besides, since dust or PM 2.5 would decrease the light intensity and shorten communicating distance, the performance of the presented smart device could be affected by smog.

This important observation authenticates the superior of the photoluminescence-enhanced Li-Fi telecommunication in long distance signal transmission. This conclusion is further confirmed by the optical images obtained through photoluminescence-enhanced Li-Fi or the traditional Li-Fi, recorded at the same conditions (Fig. S8a, b). Results shown in Fig. S8c implies that the signal transmitted by the traditional Li-Fi can be reluctantly captured by the camera when the communicating distance is at 13 m. On the contrary, the transmitted signal can be clearly identified at this distance for the PL enhanced Li-Fi (Fig. S8d). Consequently, it is reasonable to conclude that after combining the photoluminescence-enhanced Li-Fi-based telecommunication technique and a camera, the presented smart device meets one of the important criteria for remote monitoring air quality, namely, high speed transmission of the data from one site to another through electronic communication through a power-saving approach.

### Preparation of the Nanomaterial for High-Performance Sensing of Specific Air Pollutant

Beyond the power consumption, high-performance tracking of the variation of NO_2_ is another concerned issue. An effective approach to achieve the research objective is to employ specific-deigned sensing material which would be sensitive and selective to NO_2_. One of the promising candidates is ZnO, since ZnO with unique morphologies (*e.g.,* nanorods, nanosheets, nanowries, and nanoneedles) have been frequently reported to give relatively high response signal under even harsh conditions [1, 4, 27–38]. Nevertheless, most of these ZnOs suffer the problem of poor selectivity to NO_2_ and/or unstable performance at the operating temperature of higher than 300 °C, thereby hindering their broader application. In light of the fact that MOFs have been explored as promising self-sacrificial templates or precursors to construct porous oxide nanostructures with outstanding properties [39, 40], particularly, part of MOF-derived metallic oxides demonstrate unexpected sensing properties when facing diverse target analytes [40], it is expected that the self-sacrificial MOFs templates or precursors would declare a novel approach for designing ZnO with high performance in detecting NO_2_.

**Fig. 4:**
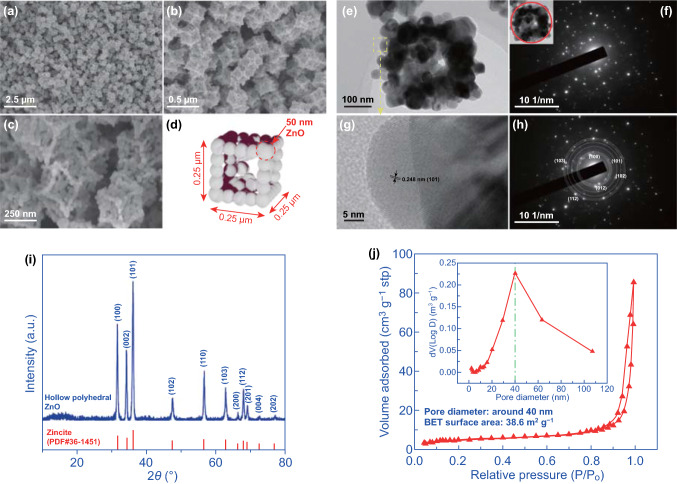
Characterization of the MOF-derived hollow polyhedral ZnO: **a–c** SEM images at different scale and **d** schema view of the MOF-derived ZnO; **e–h** TEM image and SAED pattern for the hollow polyhedral ZnO; **i** XRD pattern and **j** N_2_ adsorption–desorption isotherms as well as the pore size distributions (inset) of the hollow polyhedral ZnO

ZnO derived from the MOF precursor was synthesized and its morphology, crystal phase, and lattice parameter were investigated via the FESEM, XRD, and TEM. The high-magnified SEM images shown in Fig. 4a-c indicate the formation of hollow polyhedral ZnO after removing the MOF template at high temperature. Besides, it is found that the uniform framework (length × width × height: 0.25 × 0.25 × 0.25 μm^3^) of the hollow polyhedral ZnO is constituted by the nano-sized ZnO particles with a diameter of 50 nm. A schematic view is depicted in Fig. 4d to give a clear vision on the microstructure. Typically, the nano-sized ZnO particles acted as the “brick” and self-assembled the hollow polyhedral ZnO. Moreover, due to the interspace between each ZnO nanoparticle, a porosity structure is expected for the formed hollow polyhedral ZnO which can be confirmed in the following measurement. The nano-assembled microstructure was further investigated by HRTEM (Fig. 4e). As can be seen the interconnected nano-sized ZnO particles formed a central hollow polyhedral framework for the MOF-derived ZnO. Furthermore, selected-area electron diffraction (SAED) pattern (Fig. 4f) recorded a set of spots that implies the polycrystalline structure for the obtained hollow polyhedral ZnO. The lattice spacing estimated from Fig. 4g, h is around 0.248 nm, which can be assigned to the standard value of interplanar distance for the (101) plane (0.248 nm). Figures 4i and S9 show the XRD patterns and EDS elemental analysis for the sample. Obviously, all the diffraction peaks for the hollow polyhedral ZnO can be perfected assigned to the zincite phase (PDF# 36-1451); additionally, the EDS elemental analysis suggests no evidence of impurities left in the oxide. Because the BET surface area and porous diameter directly determine the reaction sites and gas diffusion rate, the parameters of the synthesized hollow polyhedral ZnO sample were further characterized by using nitrogen sorption technique, shown in Fig. 4j. According to the Brunauer–Emmett–Teller (BET) equation, the specific surface area of the hollow polyhedral ZnO was evaluated to be 38.6 m^2^ g^−1^. In addition, mesoporous microstructure can be confirmed for the sample, and the pore diameter estimated in the inset of Fig. 4j is around 40 nm. Such acceptable specific surface area and mesoporous microstructure of the nanoparticle-assembled hollow polyhedral ZnO would be beneficial for the gas adsorption and diffusion processes, as well as provide adequate reaction sites.

### Evaluating the Sensing Characteristics of the Standalone–Like Smart Device

**Fig. 5:**
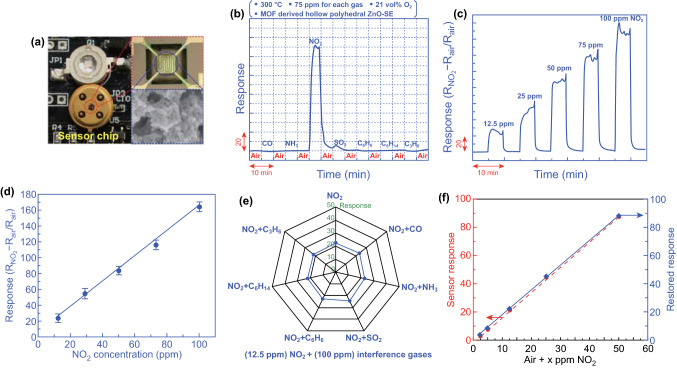
Sensing characteristics of the smart device: **a** Photography of the sensor chip made by photolithography technique; **b** Cross-sensitivity, **c** dynamic response-recovery variation, and **d** dependence of response signal on the NO_2_ concentration of the MEMS sensor consisting of the hollow polyhedral ZnO, operated at 300 °C. **e** Comparison of the sensing behavior to NO_2_ against other interference gases for the smart device, recorded at light on. **f** Consistence of the response signal that is generated or restored by the smart device or signal processing terminal to the gas mixture. The gas mixture is defined as the air mixed with x ppm NO_2_ in which x ranges from 2.5 to 50 ppm

Since the porous structure of the hollow polyhedral ZnO could accelerate the gas diffusion and offer more reaction sites, desirable NO_2_ sensing properties are speculated. Initially, the cross-sensitivity of a MEMS-type chemiresistance nanosensor based on the hollow polyhedral ZnO was evaluated with or without UV illumination (Fig. 5a), by recording the response signal with turning the LED lamp on (light on) or off (light off). It was found that the UV light emitted from the mini LED light apparently enhanced the response value of the sensor to NO_2_ and gave minor effect on the response signal of other examined gas specifies (Fig. S10). Then, the operating temperature is optimized to obtain satisfactory response/recovery rate and the largest response magnitude. Figure S11 gives the variation of response magnitude and 90% response/recovery time on the operating temperature in the range of 200–400 (with interval of 50 °C). In brevity, with an increase in the operating temperature, quick response/recovery rate was witnessed for the MEMS sensor. On the contrary, its response magnitude reached the maximum value at the operating temperature of 300 °C and started to decrease when the temperature further increased. Hence, in consideration of the largest response signal and optimal 90% response/recovery time, the operating temperature of 300 °C was selected as the optimal operating temperature and fixed for all the following research. After that, the sensing properties of the smart device integrated with the MEMS sensor were systematically studied and summarized in Fig. 5b-d. Typically, the smart device integrated with the MEMS sensor using the hollow polyhedral ZnO exhibited high selectivity to NO_2_ when against other examined air pollutants or exhaust gases (Fig. 5b), especially being illuminated by the LED lamp. The response signal ((*R*_NO2 _− *R*_a_)/*R*_a_) of the sensor to 100 ppm NO_2_ is about 164 and the corresponding 90% response/recovery time at the optimal operating condition (with UV illumination) is 52 and 50.5 s, respectively. As for other examined interference gases, a negligible response signal (within 5) was given by the created device, even been UV illuminated. Figure 5c, d shows the dynamic response–recovery curve and dependence of response signal on the concentration of NO_2_ in the range of 12.5–100 ppm. Acceptable response/recovery rate and linear relationship between the response signal and NO_2_ concentration (within the tested range) can be witnessed. Besides, the detection limit of the smart device estimated from the function of response signal vs. NO_2_ concentration (Fig. 5d) is ~ 896 ppb. To further investigate the anti-interference, response fluctuation for the smart device that is exposed to various gas mixture (12.5 ppm NO_2_ + 100 ppm interferences gases) is recorded and presented in the form of radar map (Fig. 5e). Minor fluctuated response signal that is within the acceptable range (response value: 23.24–25.68 with deviation of around 10.5%) is observed, indicating a desirable selectivity to NO_2_.

Finally, the created smart device was tested in a simulated environment for 1 day. A portable high-resolution camera was employed to capture the Li-Fi signal and a laptop serves as the data processing terminal (Fig. S12). NO_2_ in the range of 2.5–50 ppm was prepared to act as the air pollutant and directly flowed to the smart device. A 40 W fluorescent lamp was employed to supply the “sunlight.” The fluorescent lamp was kept half day light on and half day light off to simulate the daytime and nighttime. In this case, whether the device can operate at the intermittent mode for whole day without battery replacement can be confirmed. Initially, the deviation between the response signal generated by the smart device and the data restored by the data processing terminal are investigated. As shown in Fig. 5f, response signal generated by the created device perfectly matched with that of the value restored by the data processing terminal. Hence, high accuracy in real application is confirmed for the presented air quality remote tracking system. Supplementary Table 1 demonstrated that the data recorded for the device operated at the simulated daytime and nighttime for 1 day. It can be summarized that the device has shown acceptable repeatability (with deviation less than 8.9%) in sensing NO_2_ within the examined range. Furthermore, the device still revealed desirable sensing performance, even operated at the simulated nighttime. Since, the energy volume of mini battery could only support the smart device continuous operating for around 120 min, it is reasonable to conclude that the extra energy for supporting the rest of 60 min working hours (per day) is harvested from the ambient light.

Stability is a concerned parameter, particularly, when the device is being used for real application. Thus, the smart device continuously tested its sensing behavior for more than 3 months (Fig. S13). Slight decline in the response signal was observed in the first 2 weeks while after aging for 2 weeks the device gave relative stable sensing performance with an average response value of 159.2 which indicates acceptable durability to NO_2_ within the examined period. Nevertheless, in light of the fact that lifetime of the metal oxide-based sensing materials is typically within 1 year, the expected duration for the presented device is around 1 year. Based on these pilot results, it can be concluded that the smart device integrated with the MEMS sensor using hollow polyhedral ZnO derived from MOF precursor would be a promising candidate for monitoring the NO_2_ content in ambient air. These important findings confirmed the success of creating the expected standalone-like smart device for high performance and remote tracking target gas molecular which would provide a new strategy to design the WSNs for air quality monitoring.

## Conclusions

A standalone-like smart device that can remotely track the variation of air pollutants is reported. With the integration of a MEMS nanosensor that uses MOF-derived hollow polyhedral ZnO, LED lamp, mini battery, MCU, and a Si solar cell, the tailor-made smart device offers the capability of highly selective and sensitive sensing of air pollutants (e.g., NO_2_) and keep operating without battery replacement at the intermittent mode. Besides, the proposed photoluminescence-enhanced Li-Fi telecommunication technique supports the level of air pollutants to be remotely tracked in a power-saved way. By combining web camera and/or camera-loaded patrol car, the created device is expected to be useful to construct high-performance and low-power consumption air quality WSNs.

We envision that the working principle of the presented smart device could also be applied to remotely monitor other gas molecular through integrating the MESM nanosensors based on other sensing materials. Although some technical issues, for example how to harvest energy at the rainy day, how to get rid of negative impact on the circuit board that derived from moisture, have to be addressed before employing the smart device in real application, we anticipate that our reported standalone-like smart device would sever as a powerful sensing platform in preventing air pollutant-induced diseases via a more effective and low-cost approach.

## Electronic Supplementary Material

Below is the link to the electronic supplementary material.

Supplementary material 1 (PDF 1262 kb)
