# Supplementary material for: Remote Tracking Gas Molecular via the Standalone-Like Nanosensor-Based Tele-Monitoring System
Source: Nanomicro Lett. 2021 Jan 4;13:32. doi: 10.1007/s40820-020-00551-w (PMC8187508; doi:10.1007/s40820-020-00551-w)
Supplement: Supplementary file 1 — Supplementary material 1 (PDF 1262 kb) [file 40820_2020_551_MOESM1_ESM.pdf]

Supporting Information for

Remote Tracking Gas Molecular via the Standalone-Like Nanosensor -Based Tele-Monitoring System

Han Jin[1, 2, †, *], Junkan Yu[3, †], Daxiang Cui[1, 2, †], Shan Gao[4, †], Hao Yang[4, †], Xiaowei Zhang[3, †], Changzhou Hua[3, †], Shengsheng Cui[1], Cuili Xue[1], Yuna Zhang[1], Yuan Zhou[1], Bin Liu[1], Wenfeng Shen[5], Shengwei Deng[6], Wanlung Kam[7], Waifung Cheung[7]

[1]Institute of Micro-Nano Science and Technology, School of Electronic Information and Electrical Engineering, Shanghai Jiao Tong University, Shanghai 200240, People's Republic of China

[2]National Engineering Research Center for Nanotechnology, Shanghai, 200241, People's Republic of China

[3]School of of Electrical Engineering and Computer Science, Ningbo University, Ningbo 315211, People's Republic of China

[4]State Key Laboratory of Pathogen and Biosecurity, Institute of Microbiology and Epidemiology, Academy of Military Medical Sciences, Beijing 100071, People's Republic of China

[5]Ningbo Materials Science and Technology Institute, Chinese Academy of Sciences, Ningbo 315201, People's Republic of China

[6]College of Chemical Engineering, Zhejiang University of Technology, Hangzhou 310014, People's Republic of China

[7]Qi Diagnostics Ltd, Hongkong, People's Republic of China

†These authors contributed equally to this work: Han Jin, Junkan Yu, Daxiang Cui, Shan Gao, Hao Yang, Xiaowei Zhang, Changzhou Hua

*Corresponding author. E-mail: jinhan10@sjtu.edu.cn (Han Jin)

Supplementary Figures and Table

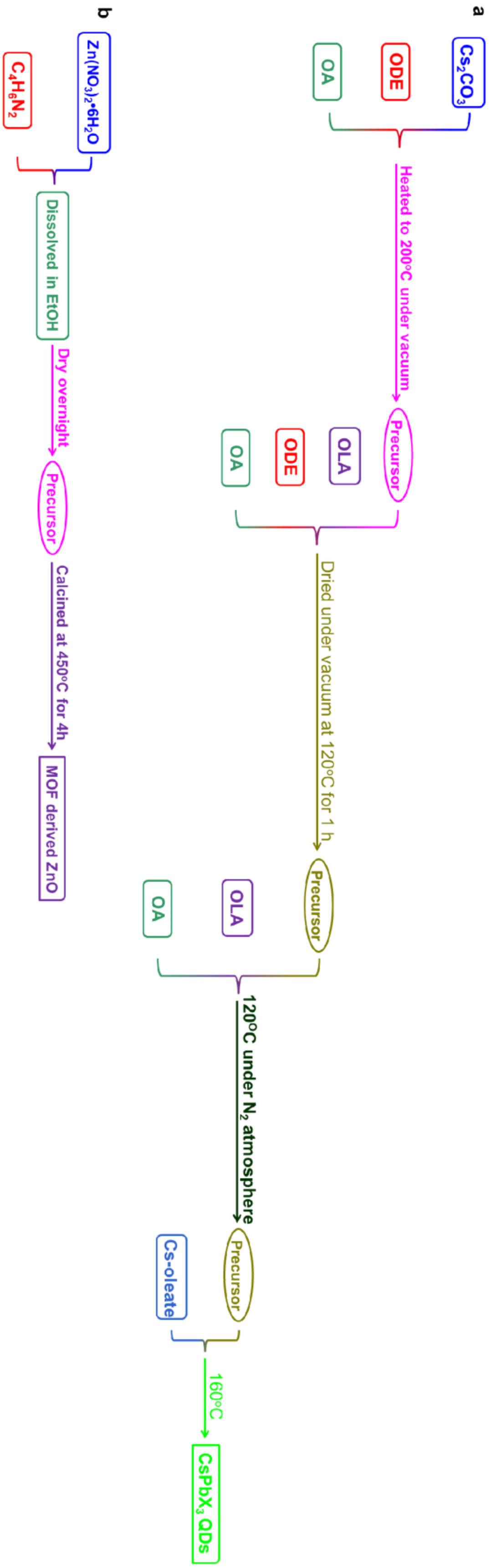

Fig. S1 Flow chart of synthesis **a** $CsPbX_3$ QDs and **b** MOF derived ZnO

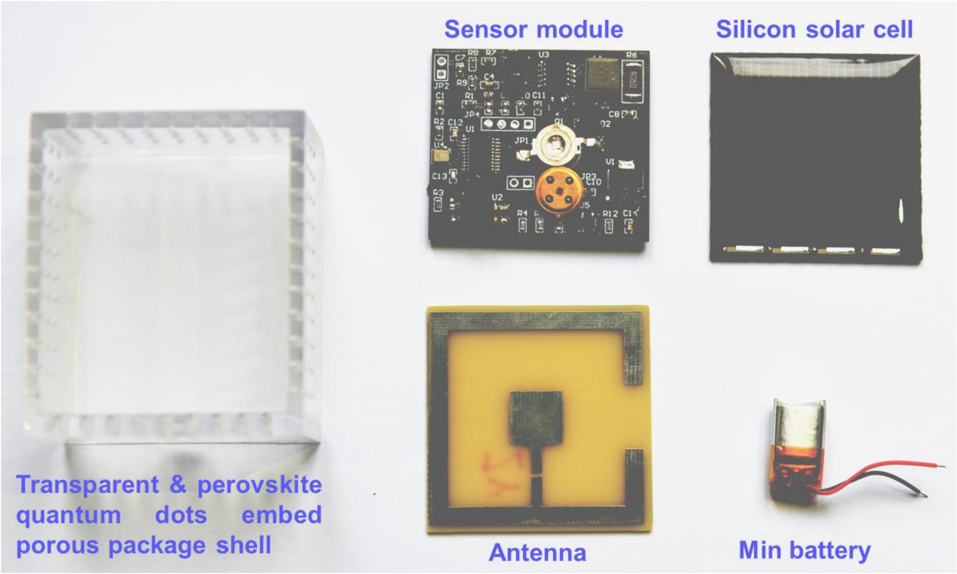

Fig. S2 Photographic image of each functional unit

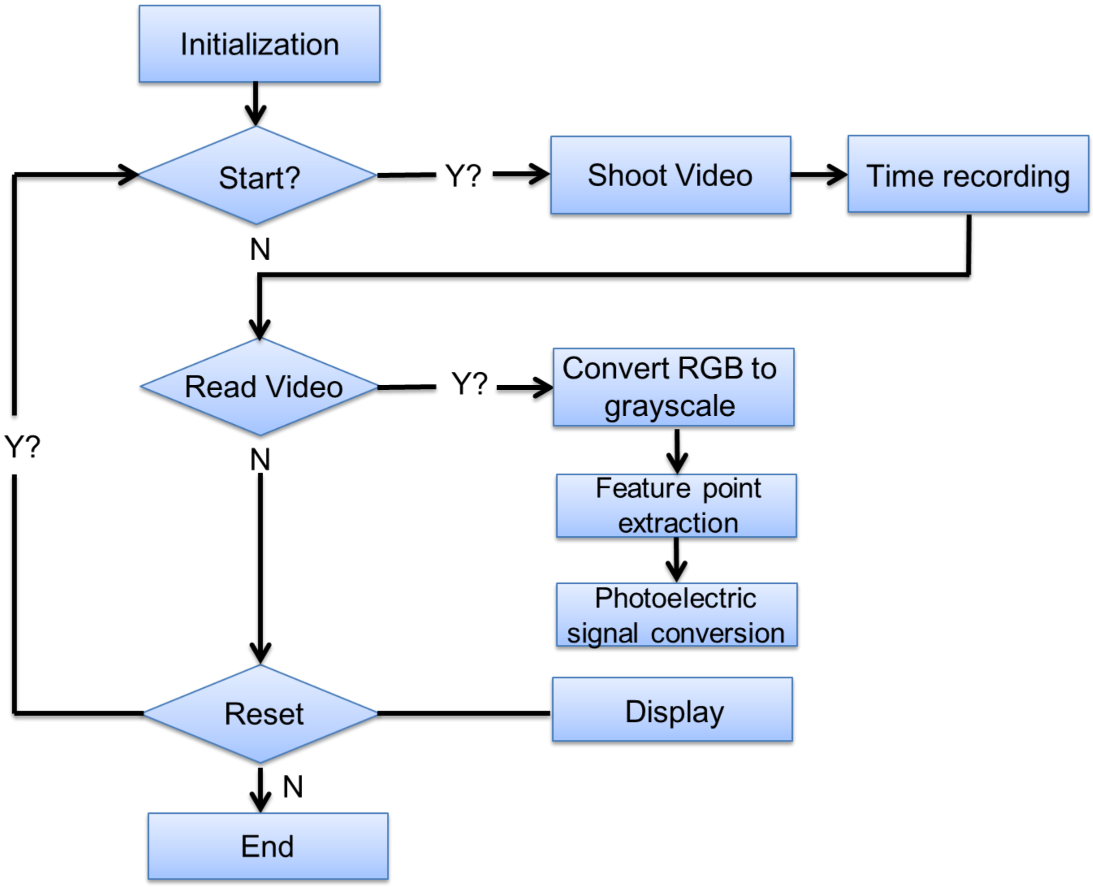

Fig. S3 Algorithm flow chart of the Li-Fi communication in which a high-resolution camera is used to capture the signal transmitted by the standalone-like smart device

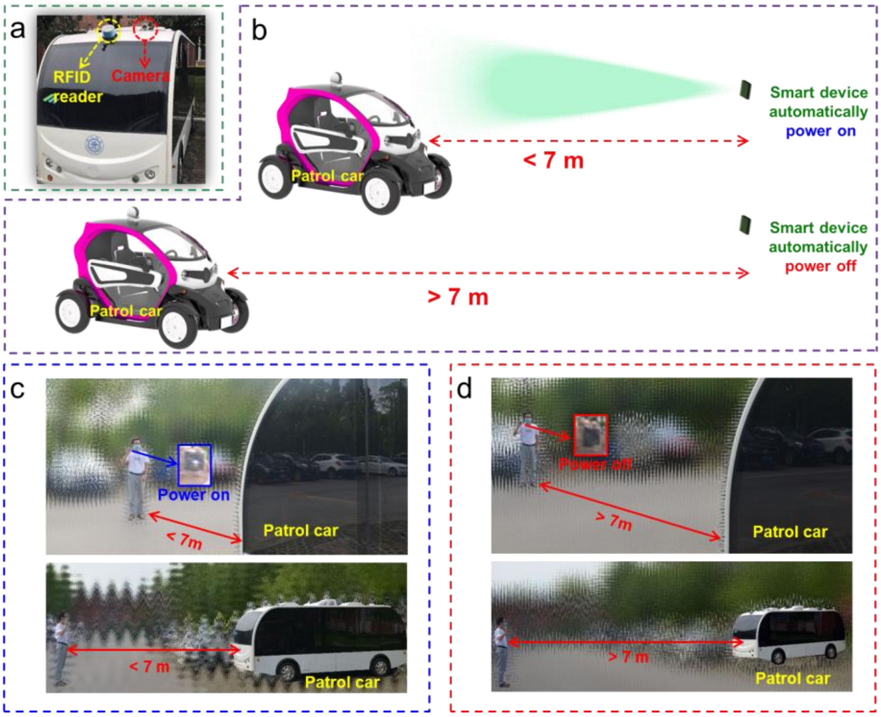

Fig. 4 a Photograph of the patrol car that loaded with RFID reader and camera; **b.** illustration of the smart device operated at awake mode; **c, d.** Demonstration of the smart device operated at the awake mode

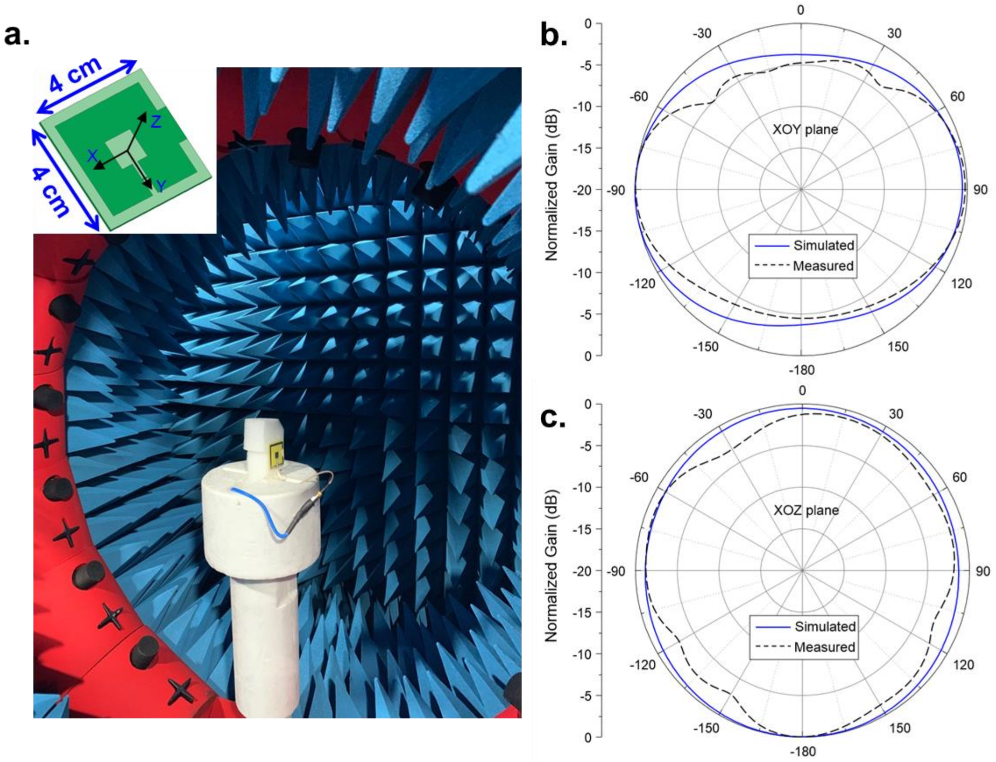

Fig. S5 a Photographic image of the set-up to characterize the antenna that integrated in the smart device; **b, c** the simulated radiation patterns of the flexible antenna measured at XOY or XOZ plane

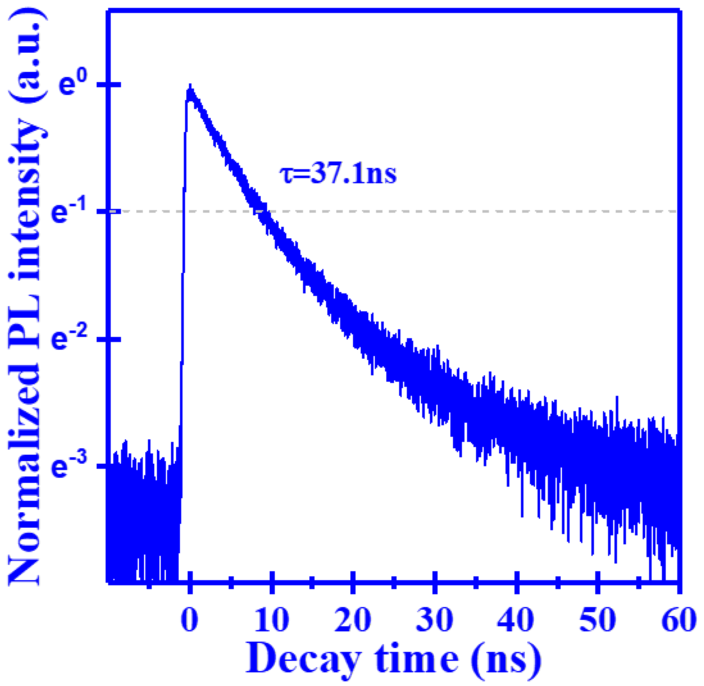

Fig. S6 Lifetime of the $CsPbCl_3$ QDs induced photoluminescence

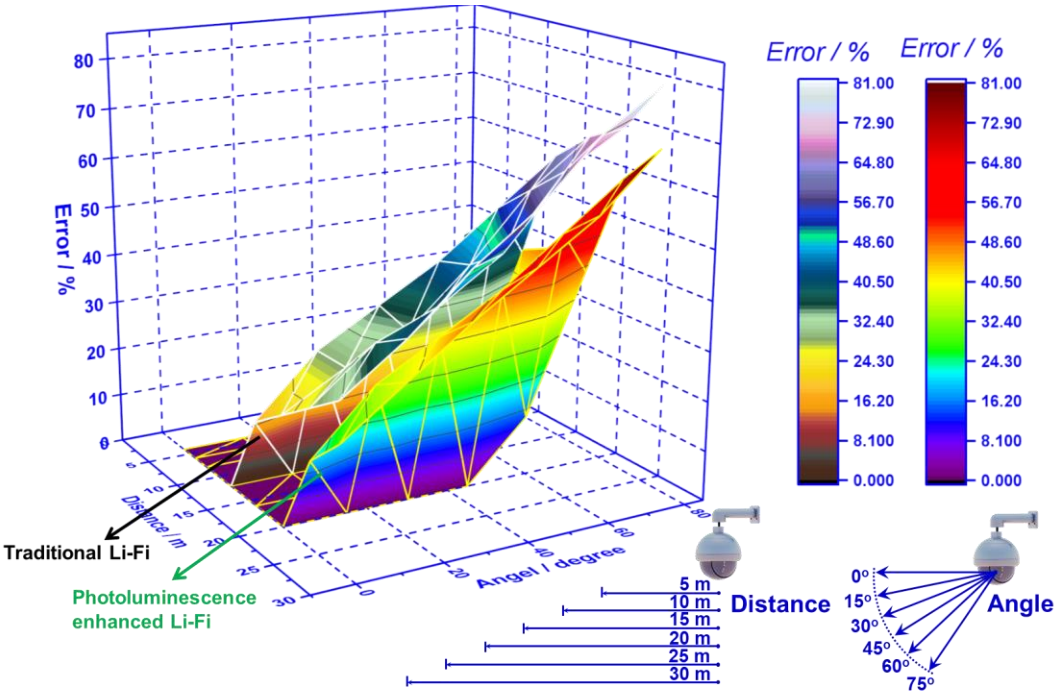

Fig. S7 Error analysis for the camera captured signal, at the viewing angel of 0-80° and the viewing distance of 0-30 m

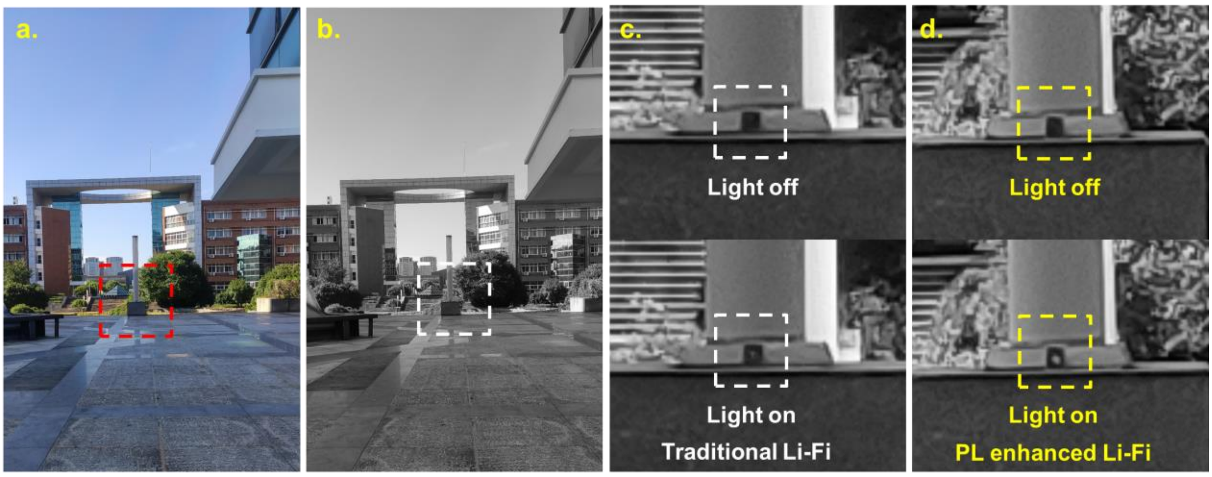

Fig. S8 a Photographic image captured by the camera; **b** Photographic image processed by the image recognition algorithm; **c** Signal transmitted by traditional Li-Fi; **d** Signal transmitted by photoluminescence (PL) enhanced Li-Fi

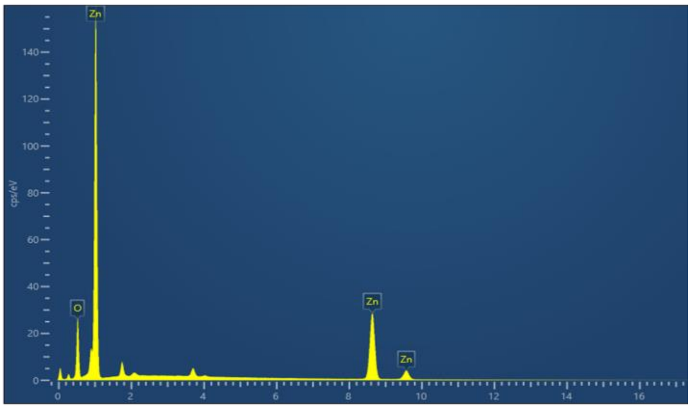

Fig. S9 EDS elemental analysis of the MOF derived ZnO

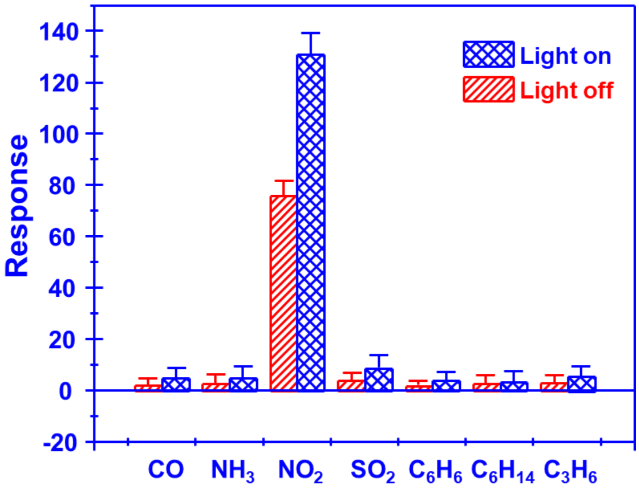

Fig. S10 Cross-sensitivity of the MEMS nanosensor that using hollow polyhedral ZnO, recorded at light on or off

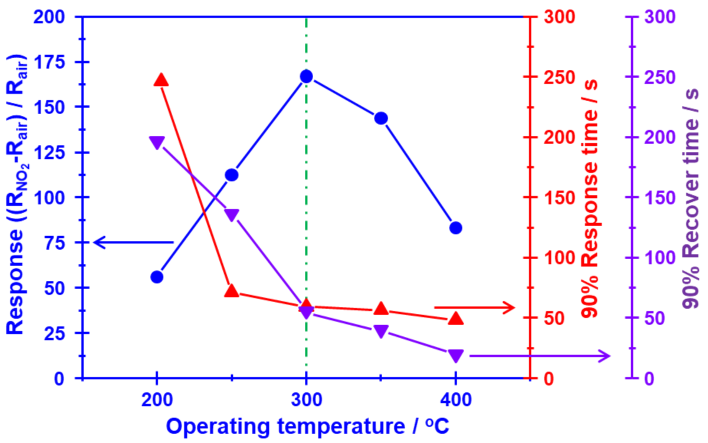

Fig. S11 Variation of the response magnitude and 90% response/recovery time on the operating temperature

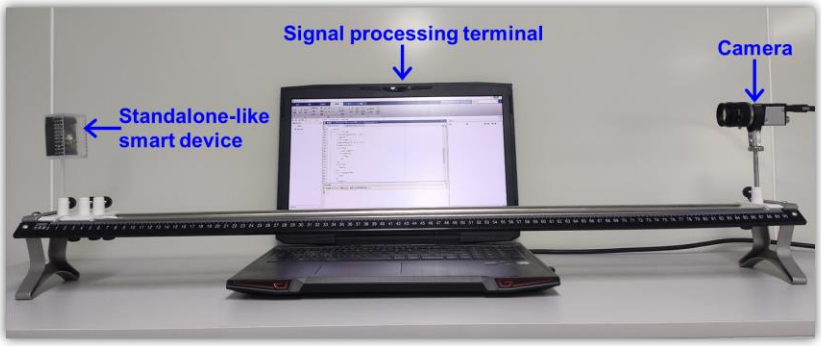

Fig. S12 Experimental set-up to simulate the remote tracking the variation of air pollutant

Table S1 Sensing characteristics of the created smart device to NO_2 in the range of 2.5-50 ppm, operated at the intermittent mode

Measurement condition	No.	Response				
		2.5 ppm	5 ppm	12.5 ppm	25 ppm	50 ppm
Fluorescent lamp light on (simulated daytime)	1	3.64	9.68	22.10	47.20	90.10
	2	3.67	9.65	21.50	48.80	89.80
	3	3.42	9.82	21.70	47.50	91.00
	4	3.57	9.65	22.50	47.40	89.40
	5	3.40	9.55	22.40	47.40	89.10
	6	3.75	9.69	22.10	47.50	90.70
Fluorescent lamp light off (simulated nighttime)	7	3.68	9.60	22.20	47.30	89.20
	8	3.90	9.57	20.90	47.80	90.50
	9	3.74	9.66	23.50	48.90	91.10
	10	3.97	9.56	22.60	47.20	91.10
	11	3.68	9.45	21.90	48.90	91.20
	12	3.44	9.71	21.20	46.00	90.90

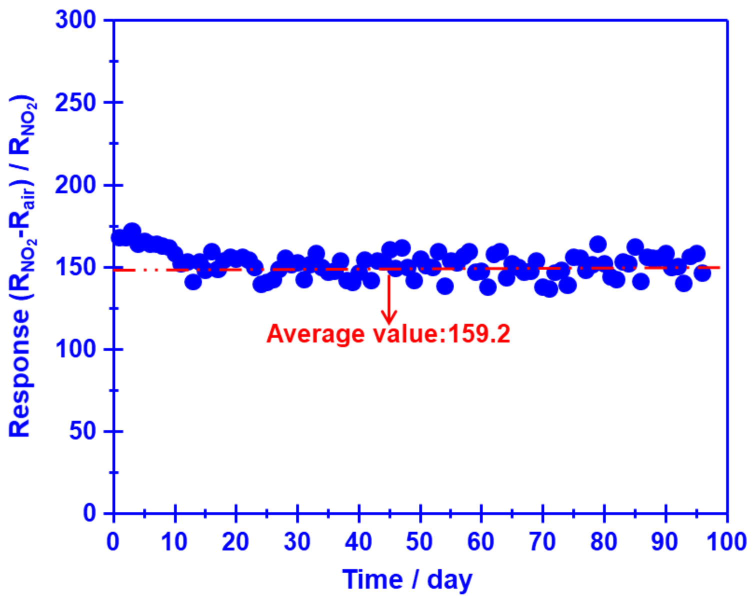

Fig. S13 Stability of the smart device consisting of the MEMS nanosensor that using MOF-derived hollow polyhedral ZnO, operated at 300 ºC for 95 days
